# Cortical morphometry in anorexia nervosa: An out‐of‐sample replication study

**DOI:** 10.1002/erv.2686

**Published:** 2019-06-06

**Authors:** Jenni Leppanen, Felicity Sedgewick, Valentina Cardi, Janet Treasure, Kate Tchanturia

**Affiliations:** ^1^ Institute of Psychiatry, Psychology and Neuroscience, Department of Psychological Medicine King's College London London UK; ^2^ South London and Maudsley Mental Health NHS Foundation Trust Section of Eating Disorders London UK; ^3^ Department of Psychology Illia State University Tbilisi Georgia

**Keywords:** anorexia nervosa, cortical folding, cortical surface area, cortical thickness, replication study

## Abstract

**Background:**

Acute anorexia nervosa (AN) is frequently accompanied by reduced global cortical volume. Investigations of local cortical alterations in AN have revealed widespread reduction in cortical thickness, gyrification, and curvature. The aim of the present study was to combine data from two previous studies to replicate previous findings.

**Methodology:**

Magnetic resonance imaging (MRI) images from 46 adult women with AN and 54 age‐matched healthy comparison (HC) women were analysed using FreeSurfer. Group differences in cortical volume and local cortical measures, including gyrification, curvature, thickness, and area, were examined controlling for dataset and age.

**Results:**

The AN group had reduced global cortical volume relative to the HC group. The AN group also had reduction in local cortical gyrification, small localised clusters of reduced cortical thickness, in the occipital and parietal cortices, and surface area in the frontal and temporal cortices. The AN group also had increased cortical thickness in the ACC relative to the HC participants.

**Conclusions:**

The present findings replicate and validate previous findings of reduced global cortical volume and local gyrification in acute AN. The findings highlight the need for further investigation of local cortical folding, thickness, and surface area in AN to gain further insight into the biological underpinnings of AN.

## 1 INTRODUCTION

Anorexia nervosa (AN) is a severe eating disorder of unknown aetiology characterised by refusal to maintain healthy body weight resulting in severe malnutrition (American Psychiatric Association, 2013). Treatment of AN remains a significant challenge with only 46% of adult AN patients reaching full recovery (Steinhausen, 2002, 2009). This illness profile has been suggested to be linked to neuroprogressive changes that develop over time and serve to perpetuate the illness (Treasure, Stein, & Maguire, 2015). These neuroprogressive changes have been suggested to be one of the reasons why recovery remains a significant challenge in the treatment of AN (Treasure et al., 2015). Thus, there has been great deal of interest to further investigate brain alterations in AN to improve the understanding of the complex biological processes that may underlie illness development and progression.

A steady accumulation of evidence shows that people with acute AN have significantly reduced global brain volume relative to age‐matched healthy comparison (HC) participants (Seitz, Herpertz‐Dahlmann, & Konrad, 2016). However, several cross‐sectional studies have suggested that this may improve with recovery, reporting no significant differences in global grey matter volume between people who have recovered from AN and healthy individuals (Bang, Rø, & Endestad, 2016; Nickel et al., 2018; Seitz et al., 2016). Reduced cortical volume has also been found to be correlated with body mass index (BMI) upon admission to inpatient treatment for AN as well as predicting BMI trajectory at 1‐year follow‐up (Seitz et al., 2015). These findings suggest that alterations in global grey and white matter volume may be not only linked to illness state and severity, but also outcome.

It has also been suggested that there is a geometric relationship between global cortical volume and local cortical thickness and surface area (Winkler et al., 2010). Local cortical thickness and surface area are believed to be linked to different aspects of cortical architecture, representing the height and number of ontogenetic columns in the neocortex, respectively (Panizzon et al., 2009; Winkler et al., 2010). This has led to increasing interest to explore whether global grey matter alterations in AN may be associated with localised cortical anomalies. A number of studies have documented widespread reduction in local cortical thickness in acute AN affecting up to 85% of the cortex (Bernardoni et al., 2016; King et al., 2015; Lavagnino et al., 2016; Miles, Voineskos, French, & Kaplan, 2018). As with cortical volume, the large‐scale reduction in cortical thickness in the acute state of illness has been found to improve significantly with even partial weight restoration (Bernardoni et al., 2016). To our knowledge, only one study has examined anomalies in cortical surface area in AN (Miles, Voineskos, et al., 2018), which reported no significant differences between women with AN and healthy women. Taken together, these findings suggest that alterations in cortical volume in AN could be related to reduced thickness rather than anomalies in surface area and be strongly linked to illness state.

Recently, there has been increasing interest in differences in localised cortical folding in AN. Cortical folding is believed to have evolved to enhance and aid cortical connectivity (Klyachko & Stevens, 2003; Mota & Herculano‐Houzel, 2012) and could thus provide further, unique insight into cortical anomalies in AN. Local indices of cortical folding are commonly measured by calculating the curvature of sulci and gyri (Dale, Fischl, & Sereno, 1999). Alternatively, one can calculate local gyrification index (LGI), which provides information about the amount of cortex that is buried inside the sulci (Schaer et al., 2012). Both approaches have been previously used to examine cortical folding in acute AN, with two studies reporting significantly increased cortical curvature in AN (Bernardoni et al., 2018; Schultz et al., 2017) and another two reporting large‐scale reduction in local gyrification (Bernardoni et al., 2018; Favaro, Tenconi, Degortes, Manara, & Santonastaso, 2015). Taken together, these seemingly contradictory findings suggests increased sharpness of sulci and gyri in acute AN resulting in reduced cortical area inside the sulci. Moreover, these alterations in cortical folding have been suggested to be related to illness state in people with AN improving significantly with weight restoration (Bernardoni et al., 2018; Favaro et al., 2015).

The aim of the present study was to validate and replicate previous findings of various cortical alterations in a larger sample of adult women with AN by combining data from two previous studies (Fonville, Giampietro, Williams, Simmons, & Tchanturia, 2014; Leppanen et al., 2017b). We examined differences between women with AN and age‐matched HC women in cortical volume as well as localised differences in cortical thickness, folding, curvature, and surface area to gain a fuller picture of anatomical differences in AN. We also investigated the extent to which any cortical alterations are related to eating disorders characteristics including self‐reported symptomatology, duration of illness, and BMI. Based on previous findings detailed above, we hypothesised that women with AN would have reduced global cortical volume as well as wide spread reduction in cortical thickness, folding, and curvature with no differences in cortical surface area. Additionally, we explored whether cortical alterations are related to eating disorder–related factors, particularly to low BMI, long duration of illness, or eating disorder symptomatology. Finally, we conducted exploratory analyses to examine the impact of age across groups and psychotropic medication within the AN group.

## 2 METHODOLOGY

### 2.1 Participants

**Figure 1:**
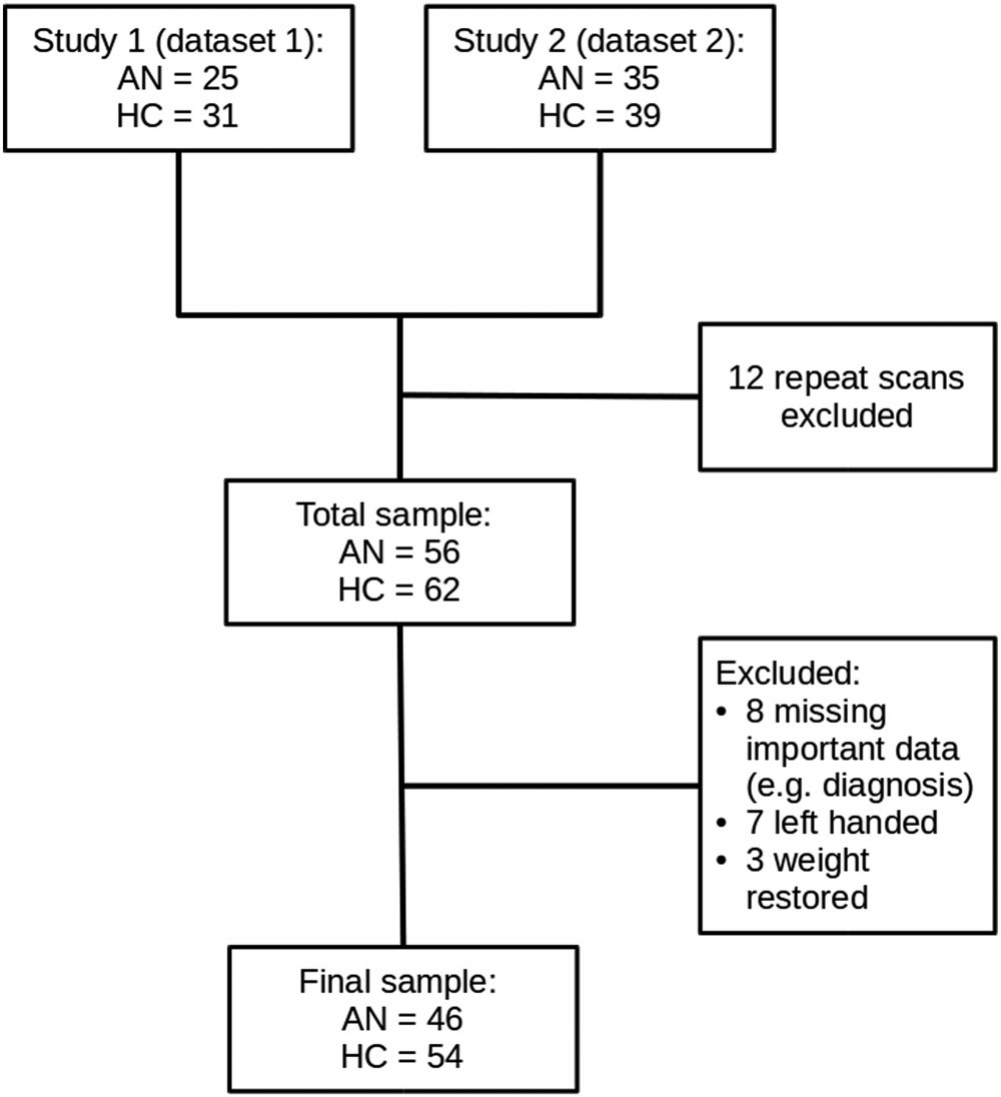
Data synthesis flow chart

The present study combined data from two previous studies (Figure 1). The studies were conducted between 2011 and 2014 and included 130 magnetic resonance imaging (MRI) scans of adult women over the age of 18 with and without AN. Twelve participants had taken part in both studies and only the first scan from these participants was included in the present study. A further 15 participants were excluded for being left handed or for missing vital data (e.g., diagnostic data), and another three AN participants were excluded for being weight restored (BMI > 18.5). The final sample consisted of 100 women, 46 participants with a current DSM‐5 diagnosis of AN and 54 healthy comparison (HC) participants. Further information about each excluded participant is given in Table S1.

The AN participants were recruited through advertisements posted at the South London and Maudsley NHS Foundation Trust and on BEAT Eating Disorders Charity website. Current DSM‐5 diagnosis of AN was confirmed with the Structured Clinical Interview for Diagnosis—Researcher version (SCID‐R; First, Williams, Karg, & Spitzer, 2015). Twenty‐three AN participants were taking psychotropic medication when they took part in the original studies. One study recorded further information about the type of psychotropic medication the participants were taking, and this information is presented Table S2. The HC participants were recruited through advertisements posted in King's College London and the local community. The HC participants were screened using the SCID‐R and were excluded if they reported current or history of psychiatric disorders. All participants were additionally screened for MRI safety and health problems other than AN. Any participant who reported being pregnant, having metal in or on the body that could not be removed, being claustrophobic, being suicidal, current or history of substance abuse or misuse, neurological disorders, or head trauma was excluded. All participants gave written and informed consent before taking part in the studies, and all study activities were conducted in accordance with the latest version of the Declaration of Helsinki (2013). Both studies were approved by National Ethics Committees (11‐LO‐0952, 11/LO/0373).

### 2.2 Procedure

Studies 1 and 2 had similar designs. Both studies took place at the Centre for Neuroimaging Sciences, King's College London, and used the General Electric Signa HDx 1.5T MR scanner. In both studies, participants' height and weight were recorded and they were asked to complete a set of self‐report questionnaires (see below) before the MRI. Study 1 was conducted between 2011 and 2014 and involved a 1‐hr MRI scan, which included a high resolution anatomical scan and three functional scans reported elsewhere (Leppanen et al., 2017a, 2017b). Similarly, the 1‐hr MRI scan conducted as part of Study 2, between 2011 and 2013, involved an anatomical scan followed by three functional scans reported elsewhere (Fonville et al., 2013; Lao‐Kaim et al., 2015; Lao‐Kaim, Giampietro, Williams, Simmons, & Tchanturia, 2014). Therefore, the anatomical data in both studies was conducted under similar settings and was regarded as comparable. Henceforth, the data from Study 1 will be referred to as Dataset 1 and data from Study 2 will be referred to as Dataset 2.

A whole brain voxel‐based morphometry analysis of data from Study 2 has been previously published (Fonville et al., 2014). However, as the present study involved combining data from Study 1 and Study 2 and examination of the cortex rather that the whole brain in an attempt to replicate findings from other laboratories, we do not believe that the present study should constitute a republication of findings.

### 2.3 Measures

As part of both studies, all participants were asked to the Eating Disorders Examination Questionnaire (EDEQ; Fairburn & Beglin, 1994). The EDEQ is a 36‐item self‐report measure that assess the presence and severity of eating disorder symptoms in the past 28 days. The total score from the EDEQ was used in the current study to investigate whether anomalies in the cortical morphometry were associated with eating disorder symptomatology. The reliability of the EDEQ in the present study was high (Cronbach's *α* = .96).

In Study 1, the presence and severity of mood and anxiety symptoms was assessed using the Hospital Anxiety and Depressions Scale (HADS; Zigmond & Snaith, 1983). The HADS is a 14‐item self‐report questionnaire that assesses the level of depression and anxiety over the past week. Study 2 used the Depression, Anxiety, and Stress Scale (DASS) to examine the presence and severity of mood and anxiety symptoms (Lovibond & Lovibond, 1995). The DASS is a 21‐item self‐report measure assessing the level of depression, anxiety, and stress over the past two weeks. The depression and anxiety subscales from both studies were converted to *z* scores in order to conduct group comparisons with the whole sample.

### 2.4 Missing data

Although participants who had important data missing, including information about diagnostic group, were excluded, some participants still had some missing data. One HC and one AN participant did not complete the EDEQ, age was not recorded from three AN participants, and duration of illness was missing from five AN participants. Additionally, more detailed information about the type of psychotropic medication 10 AN participants were taking when they took part in Study 1 was not recorded.

### 2.5 Image acquisition

Studies 1 and 2 used the same image acquisition parameters. The T1‐weighted magnetisation‐prepared gradient‐echo (MP‐ECHO) anatomical images were acquired with slice thickness of 1.2 mm, slice gap of 1.2 mm, repetition time of 8.59 s, echo time of 3.8 s, and 8° flip angle. One hundred and eighty slices were acquired to cover the whole brain. The signal was transmitted and received using an 8‐channel birdcage headcoil.

### 2.6 Statistical analysis

#### 2.6.1 Clinical and demographic data

The clinical and demographic data were analysed with R (R Core Team, 2018). The two datasets used in the analyses were first compared to ensure there were no significant differences in demographic or clinical characteristics (Table S3). Although no significant differences were present, dataset was still added as a nuisance covariate in subsequent analyses to ensure group differences were not due to small differences between the datasets. Linear regressions were used to assess differences between the groups in age, BMI, eating disorder symptomatology, depression, and anxiety with dataset as a nuisance covariate. *p* < .05 was considered significant.

#### 2.6.2 Neuroimaging data

The anatomical MRI data from the two studies were preprocessed using FreeSurfer version 6.0.0 (https://surfer.nmr.mgh.harvard.edu/). The *recon‐all ‐all* pipeline was used to conduct the initial preprocessing, which included skull stripping, volumetric registration, normalisation, volumetric labelling, segmentation, smoothing, and cortical parcellation (https://surfer.nmr.mgh.harvard.edu/fswiki/recon‐all). After the initial preprocessing, quality assessment was conducted by two of the authors (J. L. and F. S.). Each slice was visually inspected for skull stripping errors, segmentation errors, normalisation errors, pial surface errors, and topological defects following the FreeSurfer guidelines (https://surfer.nmr.mgh.harvard.edu/fswiki/FsTutorial/TroubleshootingDataV6.0). The appropriate preprocessing steps were then repeated for the participants whose images required editing. The initial preprocessing provided measures of local cortical thickness, curvature, and cortical surface area as well as a measure of global cortical volume. All thickness, curvature, and surface area images were smoothed with a 10‐mm full‐width/half‐maximum kernel.

After the initial preprocessing steps were successfully completed, further preprocessing with the *recon‐all ‐localGI* pipeline was conducted to calculate LGI (https://surfer.nmr.mgh.harvard.edu/fswiki/LGI). The LGI provides a measure of cortical folding by quantifying the amount of cortex that is hidden inside the sulci, with higher values indicating that more cortical surface is buried within the sulci. This is different from local cortical curvature, which provides information about sharpness of the sulci and gyri rather than the amount of sulcal depth. All LGI images were smoothed with 5 mm full‐width/half‐maximum kernel to avoid excessive smoothing of the LGI surface.

Following preprocessing, global cortical volume measures were extracted using the *asegstats2table* command and group differences were examined using R. Prior to examining group differences in global cortical volume, differences between the two datasets were examined using linear regression. There were no significant differences between the two datasets in cortical volume (Table S3) and thus, both datasets were entered into further analysis. A linear regression was used to asses group differences in global cortical volume. Dataset was added as a nuisance covariate to ensure any group differences that arose from the data were not due to subtle differences between the two datasets that were not apparent in the initial comparison between the two datasets. Similarly, although there were no significant differences between the groups or the datasets in age, it was added as a nuisance covariate along with estimated total intracranial volume (ICV). This was done to avoid any false findings arising from potential differences in age between the groups or datasets as age has been found to impact both global and local cortical morphometry (Ducharme et al., 2016; Fjell, McEvoy, Holland, Dale, & Walhovd, 2014; Forde et al., 2017; Gennatas et al., 2017; Klein et al., 2014; Wierenga, Langen, Oranje, & Durston, 2014; Zhou, Lebel, Treit, Evans, & Beaulieu, 2015). We also explored the impact of psychotropic medication on cortical volume within the AN group comparing AN participants who were taking medication during the time of the studies and those who were medication‐free.

Group differences in local cortical morphometry, including thickness, curvature, LGI, and surface area, were assessed in *Qdec*. As above, prior to group analysis, we ensured that there were no significant differences between the two datasets in any of the local cortical morphometry measures. Although no such differences were apparent, dataset was added as a nuisance covariate in all subsequent group comparisons to ensure significant findings were not due to subtle differences between the two datasets. Similarly, age was also added as a nuisance covariate to control for local cortical differences that could be explained by age. In the main analysis, differences in local cortical thickness, curvature, LGI, and surface area between the AN and HC groups, controlling for dataset, were examined with general linear models. Within the AN group, we also conducted further exploratory analyses to examine the impact of medication status (medication vs. medication‐free) on all measures of local cortical morphometry. Finally, we also explored the extent to which global cortical volume influenced local cortical measures within the AN group. Cluster‐wise correction for multiple comparisons was conducted with permutation tests. The initial, uncorrected cluster defining threshold was *p* < .0005, which was chosen to account for the fact that each hemisphere was analysed separately.

Correlation analyses were conducted within the AN group to examine whether anomalies in global cortical volume or local cortical morphometry were related to eating disorder characteristics, including duration of illness in years, BMI, and self‐reported eating disorder symptomatology as measured with the EDEQ. To examine correlations between local cortical morphometry and eating disorder characteristics, the cluster mean values were extracted if significant group differences in cortical thickness, curvature, LGI, or surface area were present. Correction for multiple comparisons was conducted with false discovery rate (*q* = 0.05), and *p* < .0007 was considered significant.

## 3 RESULTS

### 3.1 Sample characteristics

Demographic and clinical sample characteristics are summarised in Table 1. The groups were matched for age. As expected, there were significant differences between the groups in eating disorder characteristics and in depression and anxiety with the AN group reporting more symptoms and lower BMI.

**Table 1:** Sample characteristics controlling for dataset

	AN	HC	t statistic, p value
	Mean (SD)	Mean (SD)	
	Range	Range	
	N	N	
Age	27.51 (9.24)	26.35 (4.47)	Group: *t*(94) = 0.83, *p* = .408 Dataset: *t*(94) = −0.59, *p* = .560
	18.00–60.00	20.00–45.00	
	43	54	
EDEQ total	4.01 (1.01)	0.54 (0.51)	Group: *t*(95) = 21.73, *p* < .001 Dataset: *t*(95) = −0.05, *p* = .958
	1.50–5.82	0.00–2.55	
	45	53	
BMI	15.73 (1.41)	21.49 (1.97)	Group: *t*(97) = −7.30, *p* < .001 Dataset: *t*(97) = 0.20, *p* = .422
	12.0–18.0	18.00–26.30	
	46	54	
Depression *z* score	0.80 (0.83)	−0.77 (0.30)	Group: *t*(97) = 13.32, *p* < .001 Dataset: *t*(97) = 0.08, *p* = .939
	−0.65–3.57	−1.88–0.34	
	46	54	
Anxiety *z* score	0.84 (0.67)	−0.82 (0.39)	Group: *t*(97) = 14.38, *p* < 0.001 Dataset: *t*(97) = −0.25, *p* = .802
	−0.74–2.43	−0.99–0.21	
	46	54	
Duration of illness (years)	11.39 (9.22)	N/A	N/A
	1.00–36.00		
	41		

Abbreviations: AN, anorexia nervosa; BMI, body mass index; EDEQ, Eating Disorder Examination Questionnaire; HC, healthy comparison; N/A, not applicable; SD, standard deviation.

### 3.2 Cortical volume

**Figure 2:**
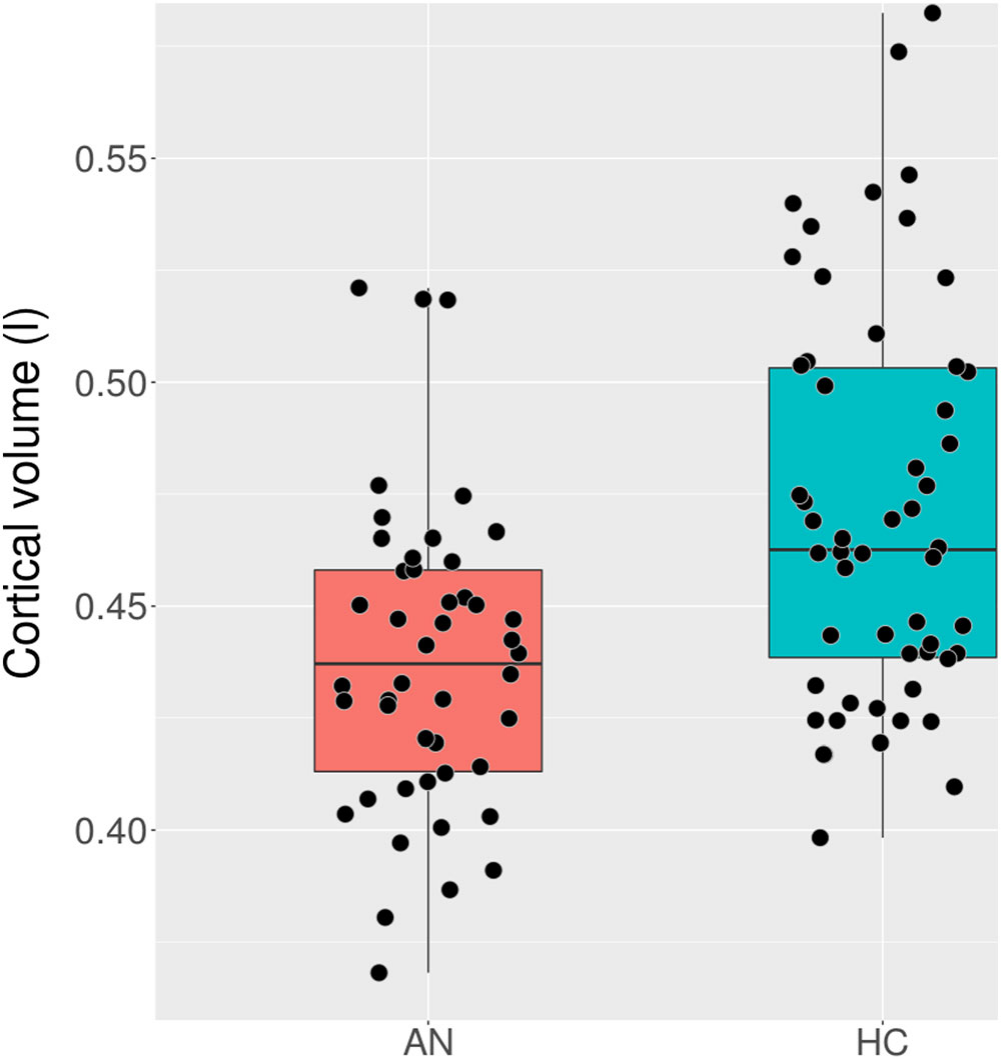
Global cortical volume in litres by group [Colour figure can be viewed at wileyonlinelibrary.com]

Group difference in global cortical volume is shown in Figure 2. Controlling for dataset, age, and estimated total ICV, there was a significant difference between the groups in global cortical volume with the AN group having significantly smaller cortex, group: *t*(92) = 4.00, *p* < .001; AN: *M* = 437,894.40 mm^3^, *SD* = 34,052.40; HC: *M* = 471,102.78 mm^3^, *SD* = 44,395.16). There was also a significant effect of age with older participants having reduced cortical volume compared with the younger participants, *t*(92) = −4.24, *p* < .001, and a significant effect of estimated total ICV indicating that those with larger ICV also had larger cortical volume, *t*(92) = 14.48, *p* < .001. There was no significant difference between the datasets, controlling for group and age, dataset: *t*(92) = −1.14, *p* = .258; dataset 1: *M* = 455,304.32 mm^3^, *SD* = 46,630.96; dataset 2: *M* = 456,416.24 mm^3^, *SD* = 39,268.11).

Following correction for multiple comparisons, the correlation analyses revealed no significant correlations between global cortical volume and eating disorder characteristics within the AN group (Table S4). The exploratory analysis also revealed that controlling for dataset, there was no significant difference in global cortical volume between AN participants who were taking psychotropic medication during the studies and those who were medication‐free (Table S5). There was also no significant difference between the datasets controlling for medication status.

### 3.3 Cortical thickness

**Figure 3:**
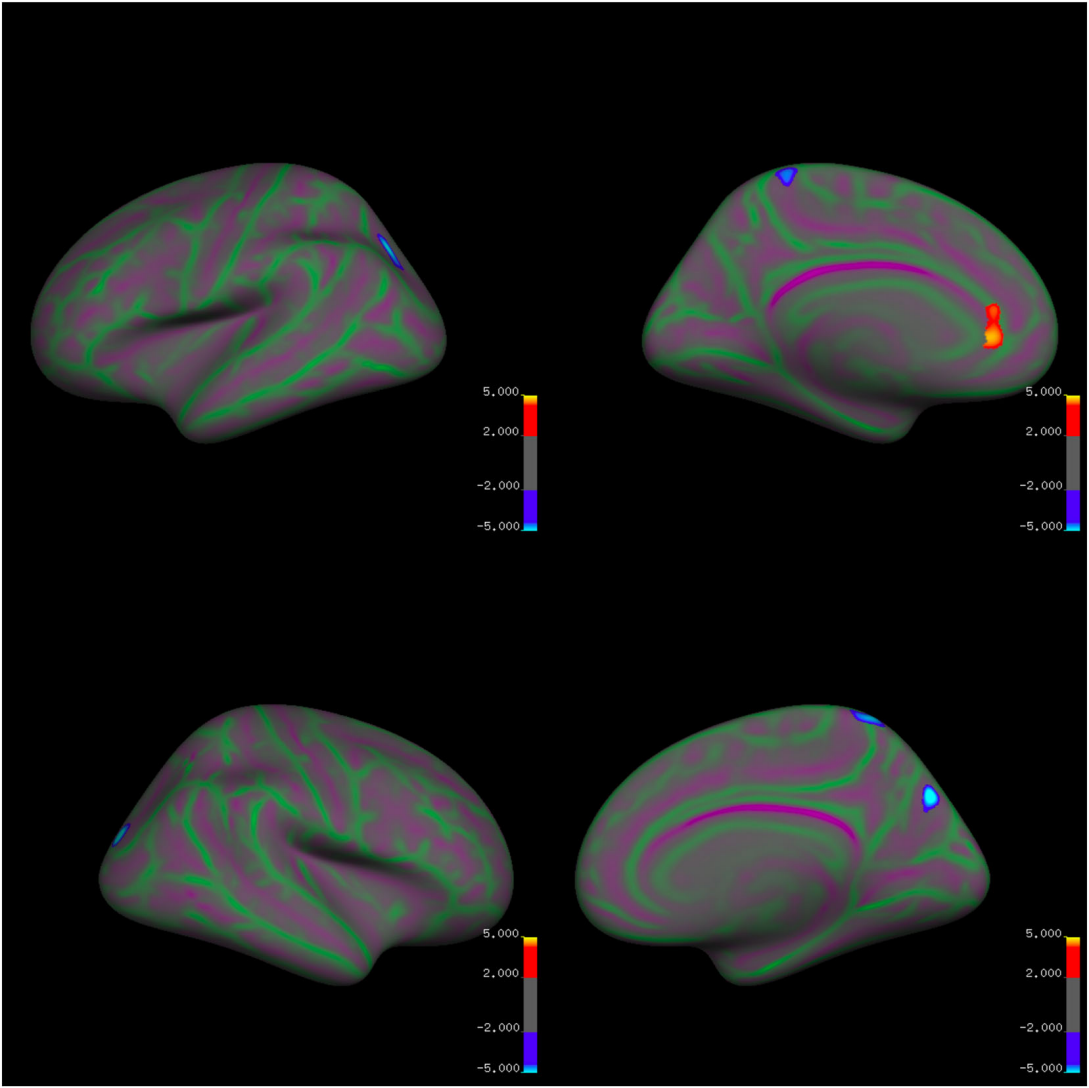
Group differences in local cortical thickness controlling for dataset and age [Colour figure can be viewed at wileyonlinelibrary.com]

While controlling for dataset and age, there were significant differences between the groups in local cortical thickness in several small clusters (Table 2, Figure 3). In the left hemisphere, relative to the HC participants, the AN group had significantly thinner cortex in a two small clusters in the superior parietal cortex and paracentral cortex. In the right hemisphere, the AN participants had significantly reduced cortical thickness in small regions in the back of the brain, including the superior parietal cortex, precuneus, and lateral occipital cortex. The AN group had significantly thicker cortex than the HC participants in a small cluster in the rostral anterior cingulate cortex.

**Table 2:** Group differences in local cortical thickness controlling for dataset

Peak MNI coordinates			Cluster z score	Corrected CWP	Cluster size (mm^2^)	Number of vertices	Peak region
X	Y	Z					
−22.9	−64.2	29.7	−5.01	0.013	168.61	392	SPC
−14.4	−41.0	67.1	−4.21	0.041	133.06	287	Paracentral cortex
−5.6	34.4	2.6	4.46	0.042	130.60	246	ACC
11.7	−52.2	64.9	−4.78	0.007	189.52	482	SPC
21.3	−88.4	18.2	−5.98	0.009	182.29	259	LOC
18.1	−70.8	35.7	−5.92	0.019	158.01	289	Precuneus

Abbreviations: ACC, anterior cingulate cortex; CWP, cluster‐wise *p* value; LOC, lateral occipital cortex; MNI, Montreal Neurological Institute; SPC, superior parietal cortex.

After correction for multiple comparisons, there were no significant correlations between the mean cluster thickness and the eating disorder variables within the AN group (Table S4). There were also no significant differences between AN participants who were taking psychotropic medication and those who were not medicated in cortical thickness.

### 3.4 Local gyrification index

**Figure 4:**
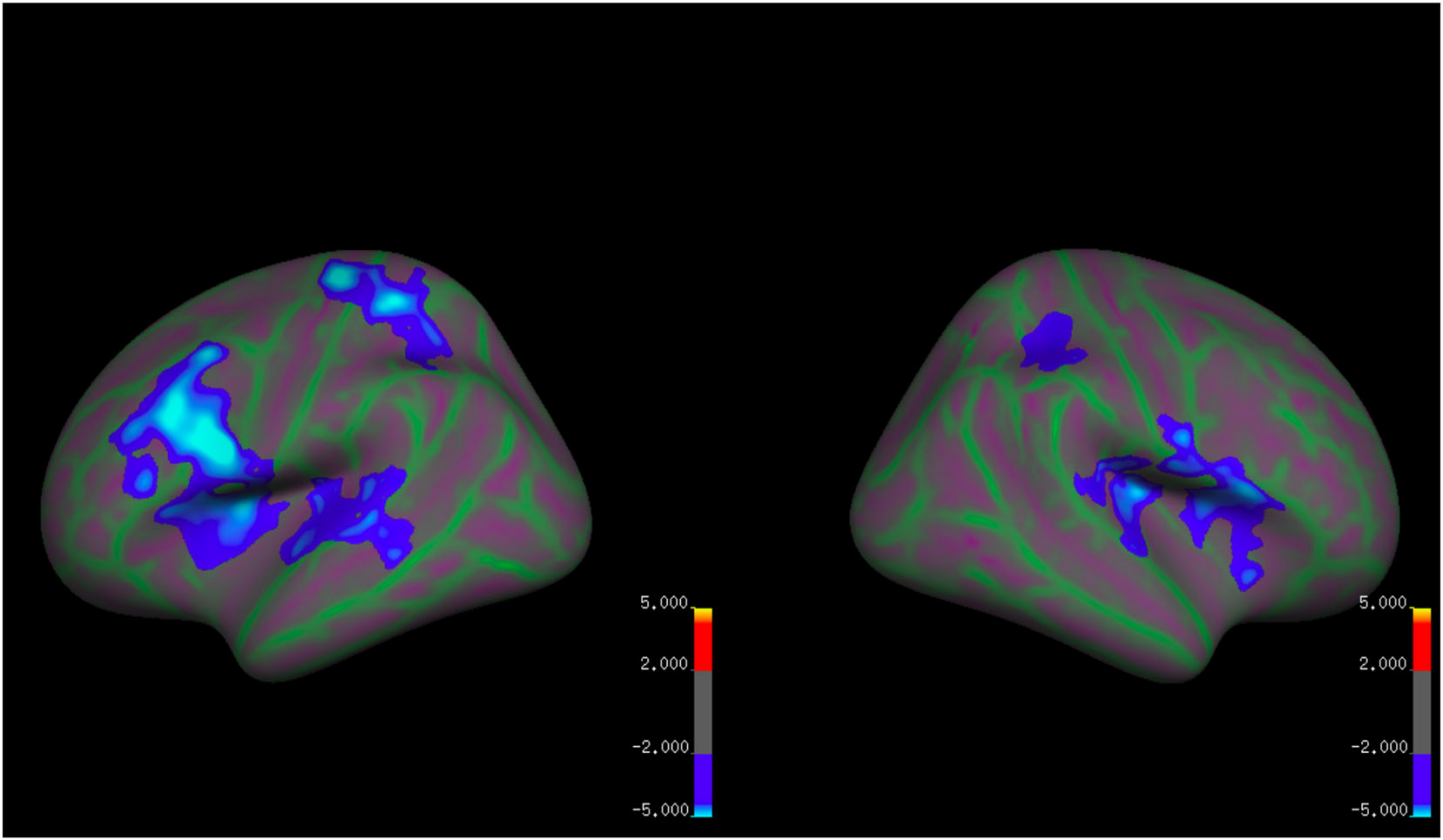
Group differences in local gyrification index controlling for dataset and age [Colour figure can be viewed at wileyonlinelibrary.com]

There were widespread differences between the groups in LGI, controlling for dataset and age (Table 3, Figure 4). Relative to the HC participants, the AN group had significantly reduced LGI in large clusters in the left hemisphere. These included a cluster in the lateral frontal cortex that extended to the insula, a cluster in the superior temporal cortex (STC), a large cluster in the postcentral cortex. Similarly, the AN group had significantly reduced LGI also in the right hemisphere, including clusters in the lateral frontal cortex that extended into the anterior and middle insula, supramarginal gyrus, and posterior insula. There were no areas of significantly greater LGI in the AN group relative to the HC participants.

**Table 3:** Group differences in local gyrification index controlling for dataset

Peak MNI coordinates			Cluster z score	Corrected CWP	Cluster size (mm^2^)	Number of vertices	Peak region
X	Y	Z					
−45.2	2.5	20.3	−6.73	0.0002	4424.48	9381	LFC
−22.9	−26.7	51.6	−5.45	0.0002	2103.19	4995	Postcentral cortex
−65.0	−31.5	4.8	−4.29	0.0002	1889.76	4483	STC
52.8	14.7	9.3	−4.57	0.0002	1930.45	4279	LFC
32.6	−22.6	15.8	−4.67	0.0002	1222.40	3267	Insula
45.5	−31.8	39.4	−3.76	0.0002	780.80	2179	SMG

Abbreviations: CWP, cluster‐wise *p* value; MNI, Montreal Neurological Institute; LFC, lateral frontal cortex; SMG, supramarginal gyrus; STC, superior temporal cortex.

Blue clusters indicate reduced LGI in the AN group relative to the HC group. Red and yellow clusters indicate reduced LGI in the HC group relative to the AN group. Green areas mark sulci and purple areas mark gyri.

In the postcentral cortex, there was a significant negative correlation between mean cluster LGI and duration of illness within the AN group (*r* = −.54, *p* = .0003). There were no further significant correlations between mean cluster LGI and eating disorder variables within the AN group after correcting for multiple comparisons (Table S4). There were also no significant differences between AN participants who were taking psychotropic medication and those who were not medicated in LGI.

### 3.5 Cortical curvature

There were no significant differences between the groups in local curvature while controlling for dataset and age. There were also no significant differences between AN participants who were taking psychotropic medication and those who were not medicated in cortical curvature.

### 3.6 Cortical surface area

**Figure 5:**
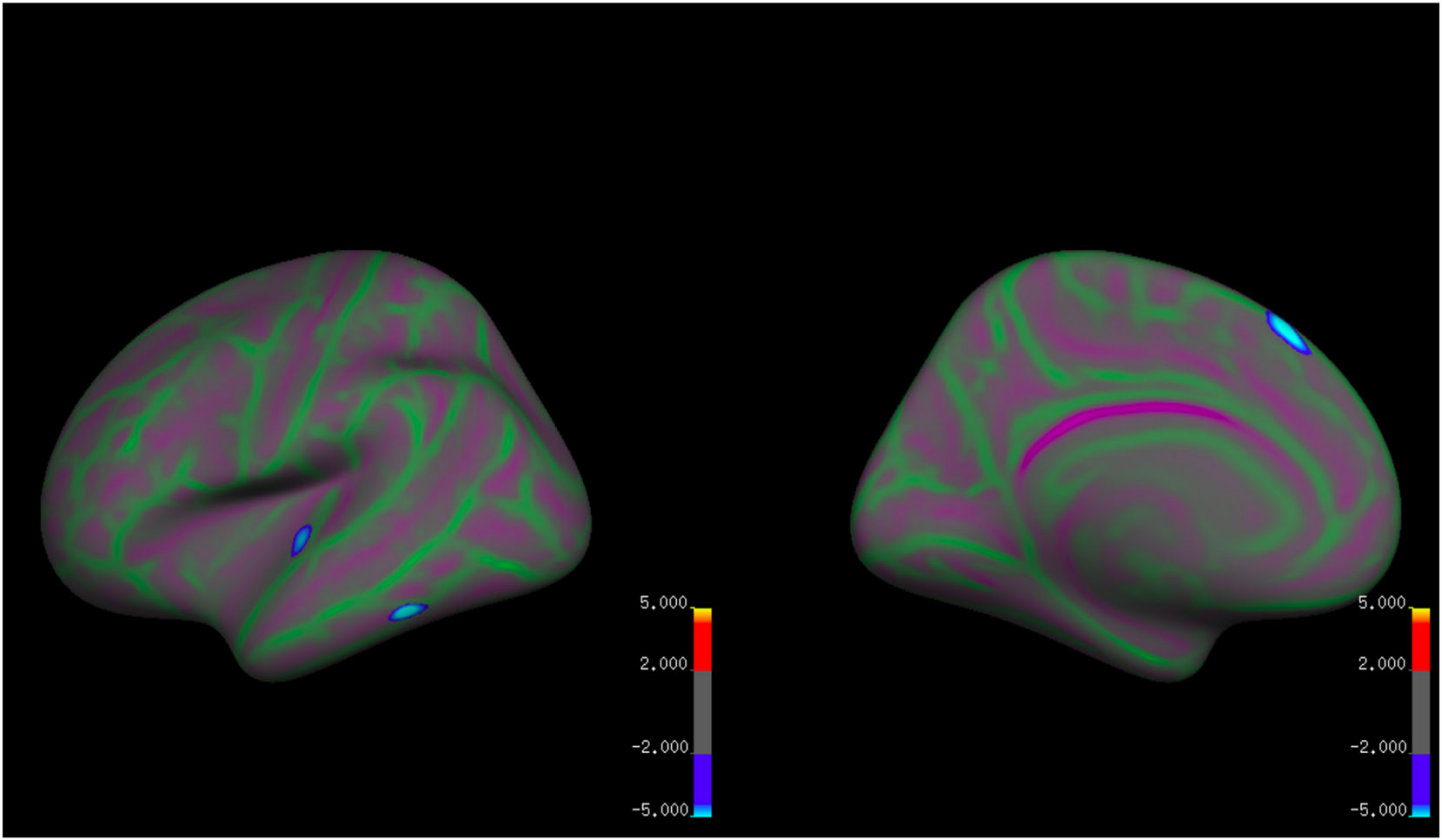
Group differences in local cortical surface area controlling for dataset and age [Colour figure can be viewed at wileyonlinelibrary.com]

There were significant differences between the groups in local cortical surface area in three small clusters in the left hemisphere controlling for dataset and age (Table 4; Figure 5). The AN participants had significantly reduced cortical surface area relative to the HC group in the superior temporal cortex, superior frontal cortex, and inferior temporal cortex. There were no regions where the AN group had significantly increased cortical surface area relative to the HC group.

**Table 4:** Group differences in local cortical surface area controlling for dataset

Peak MNI coordinates			Cluster z score	Corrected CWP	Cluster size (mm^2^)	Number of vertices	Peak region
X	Y	Z					
−6.9	27.5	53.3	−6.03	0.006	179.71	332	SFC
−56.3	−46.4	−19.7	−5.76	0.028	132.50	171	ITC
−49.2	−15.9	2.1	−4.62	0.049	113.24	241	STC

Abbreviations: CWP, cluster‐wise *p* value; MNI, Montreal Neurological Institute; ITC, inferior temporal cortex; SFC, superior frontal cortex; STC, superior temporal cortex.

Blue clusters indicate reduced cortical surface area in the AN group relative to the HC group. Red and yellow clusters indicate reduced cortical surface area in the HC group relative to the AN group. Green areas mark sulci and purple areas mark gyri.

There were no significant correlations between mean cortical surface area and eating disorder variables within the AN group after correction for multiple comparisons (Table S4). There were also no significant differences between AN participants who were taking psychotropic medication and those who were not medicated in cortical surface area.

### 3.7 Relationship between cortical volume and local measures

Within the AN group, we explored the relationship between global cortical volume and local cortical measures. Global cortical volume was positively related to cortical surface area in a small region of the right superior frontal cortex (Table S6). Cortical volume was not significantly related to other measures of local cortical morphometry.

## 4 DISCUSSION

The aim of the present study was to combine anatomical MRI data from two previous studies to replicate and validate previous findings of global and local cortical alterations in women with AN. As hypothesised, the present findings showed globally reduced cortical volume and reduced local gyrification in women with AN. However, contrary to our hypotheses, we did not find widespread reduction in local cortical thickness or curvature in AN. Instead the results showed small regions of reduced cortical thickness in the occipital and parietal cortices bilaterally in women with AN and no group differences in cortical curvature between women with and without AN. Additionally, we found significant group differences in local cortical surface area in a number of small clusters in the temporal and frontal cortices bilaterally. Finally, there was a significant correlation between local gyrification in the postcentral cortex and duration of illness.

The present study replicated and validated previous findings documenting reduced global cortical volume and reduced gyrification in the lateral frontal, temporal, and postcentral cortices bilaterally in women with acute AN. Previous work has suggested that reduced cortical volume and local gyrification in AN may be linked to illness state and improves with recovery (Bang et al., 2016; Bernardoni et al., 2018; Favaro et al., 2015; Miles, Voineskos, et al., 2018; Seitz et al., 2016). A number of cross‐sectional studies have documented that there are no significant differences in cortical volume between those who have recovered from AN and HC participants (Bang et al., 2016; Nickel et al., 2018; Seitz et al., 2016). Furthermore, a recent large‐scale longitudinal study by Bernardoni et al. (2018) found that widespread alterations in local gyrification in patients with acute AN normalised following 10% short‐term increase in BMI. One study has also reported that AN patients who had reached full recovery no longer showed reduced gyrification in the postcentral cortex relative to HC participants in cross‐sectional and longitudinal assessments unlike those who remained unwell (Favaro et al., 2015). Interestingly, the present study also found that the level of gyrification in the postcentral cortex correlated negatively with illness duration, which could be interpreted as a potential indicator of severe and enduring form of AN. Taken together, these findings appear to suggest that widespread alterations in local gyrification may be linked to the illness state in AN, but some localised differences could serve as indicators of treatment resistance or severe and enduring form of illness warranting further investigation.

The present finding did not replicate previous work that has documented widespread reduction in cortical thickness and regional increases in cortical curvature. Previous work has not only found large‐scale, global reduction in cortical thickness and increase cortical curvature in AN but also documented a close link between eating disorder characteristics and these measures of local cortical morphometry (Bernardoni et al., 2016; King et al., 2015; Lavagnino et al., 2016; Miles, Voineskos, et al., 2018). Cross‐sectional studies have found that the widespread reduction in cortical thickness evident in acute AN is no longer present in those who have recovered from AN (King et al., 2015; Miles, Voineskos, et al., 2018; Nickel et al., 2018). Reduced cortical thickness has also been linked to low BMI and increased self‐reported eating disorder symptomatology among participants with acute AN (Bernardoni et al., 2016; Lavagnino et al., 2016). These findings are solidified by longitudinal work that has reported that the globally reduced cortical thickness and increased cortical curvature appear to normalise following short‐term partial weight restoration (Bernardoni et al., 2016, 2018). In these studies, normalisation of both cortical thickness and curvature were correlated with positive changes in weight among the AN patients (Bernardoni et al., 2016, 2018). The present study, on the other hand, found only small clusters of reduced cortical thickness largely concentrated in the occipital and parietal cortices in women with AN, and no significant group differences in curvature were found. Furthermore, the mean cortical thickness differences within these regions were not correlated with BMI, duration of illness, or self‐reported eating disorder symptomatology.

Failure to replicate previous findings may be at least partly related to the age of the sample in the present study, which was on average older than the participants in some previous studies (Bernardoni et al., 2016; King et al., 2015). It has been previously shown that cortical thickness generally decreases with age (Ducharme et al., 2016; Gennatas et al., 2017; Hogstrom, Westlye, Walhovd, & Fjell, 2013). Accelerated reduction in cortical thickness during maturation from childhood or adolescence to young adulthood has also been reported (Khundrakpam, Lewis, Kostopoulos, Carbonell, & Evans, 2017; Zhou et al., 2015). Additionally, a recent review found that adolescent participants with acute AN had significantly more reduced global cortical volume than adults with acute AN (Seitz et al., 2016), which could have implications for cortical thickness. Further investigation of cortical thickness in AN across lifespan to explore potential differences in cortical development and maturation may therefore be of interest.

Interestingly, the present study also found increased cortical thickness in the left ACC in the AN group relative to the HC participants. This findings is in direct contrast with the above‐mentioned previous findings (Bernardoni et al., 2016; King et al., 2015; Lavagnino et al., 2018; Miles, Voineskos, et al., 2018). A few previous studies have reported similar thickening or increased volume of the ACC in other related disorders, such as social anxiety disorder, major depressive disorder, and posttraumatic stress disorder with depression (Brühl et al., 2014; Rauch et al., 2003; Reynolds et al., 2014). These studies have suggested that the increased cortical thickness in the ACC may be linked to attempts at compensating or coping with difficulties in emotion regulation or hypervigilance to conflict (Brühl et al., 2014; Reynolds et al., 2014). However, as the present did not find significant correlations between mean cluster thickness in the ACC and eating disorder related variable, it is difficult to ascertain the role of increase thickness in the ACC may have in AN. This is particularly complicated by findings that the ACC appears to support a multitude of different functions including various aspects in emotional processing, such as emotion regulation, cognitive processing and attention, and disturbed sleep pattern (Ochsner, Bunge, Gross, & Gabrieli, 2002; Ochsner, Silvers, & Buhle, 2012; Shackman et al., 2011; Steele & Lawrie, 2004; Winkelman et al., 2013). Therefore, further investigation of the impact of potential thickening of the cortex in this region in AN may have on brain function and clinical outcomes may be of interest.

Contrary to our hypothesis, we found significant group differences in local cortical surface area in a number of small clusters in the middle frontal and temporal cortices. This does not replicate previous work by Miles, Voineskos, et al. (2018), which to our knowledge was the first study to examine local alterations in cortical surface area in people with AN relative to HC participants and found no significant group differences. However, the study by Miles, Voineskos, et al. (2018) had a relatively small sample size, and it is, therefore, possible that these differences in findings are at least partly due to differences in statistical power. Indeed, despite the lack of group differences, the authors did find that local cortical surface area in the lateral frontal cortex and the insula correlated significantly with a standardised composite score, comprising neuroticism and attachment insecurity among those with AN (Miles, Kaplan, & Voineskos, 2018). Taken together, these findings suggest that alterations in cortical surface area may be part of the biological mechanisms that underpin AN and further work examining this is needed.

## 5 LIMITATIONS

The present study is not without limitations. First, the present study is not a true replication study, which can be defined as a study that uses the same methodology with a different sample from the same population as a previous study (Ioannidis et al., 2014; Nichols et al., 2017; Open Science Collaboration, 2015). Instead, the present study attempted to assess so‐called far replicability by using a different sample from the same population, different experimental methodology, and different analysis methods (Nichols et al., 2017). Far replicability provides information about far reaching generalisability and is often considered to the most challenging form of replication (Nichols et al., 2017). Therefore, the present study should be taken as an important step forward as it provides valuable insight into cortical morphometry in AN.

Importantly, we did not investigate differences between people with AN and HC participants in structural connectivity in the cortex, instead focusing on the macroarchitecture of the cortex. Therefore, it is difficult to ascertain whether the above‐mentioned differences between the AN and HC groups are linked to any differences in structural connections within or between these regions. Along the same lines, it is also important to note that alterations in the cortical macroarchitecture do not necessarily indicate the presence of functional differences. Therefore, further investigation of structural and functional connectivity within the cortex, as well as the degree to which any potential alterations may be linked to not only illness‐related variables but also to social, emotional, and cognitive functioning in AN, may be of interest.

The present study was cross‐sectional in nature and not a longitudinal assessment of changes in cortical morphometry in AN. Therefore, it is difficult to determine the extent to which the cortical alterations in the AN group impact illness maintenance or outcome. Furthermore, no information regarding stage of treatment was recorded to cross‐sectionally examine differences in global and local cortical morphometry across stages of recovery. Therefore, further longitudinal work that not only explores cortical macroarchitecture and connectivity and their impact on illness outcome but also aims to replicate previous longitudinal findings is still needed.

Additionally, all imaging was conducted using a 1.5‐T MRI unit. The use of higher magnetic field strength (e.g., 3.0 T) has recently become the standard, and it is possible that the lower magnetic field strength in the present study could have impacted the results. Some studies have reported that 3.0‐T MRI units have greater sensitivity to detect small scale structural alterations (Chow et al., 2015; Stankiewicz et al., 2011). However, a recent review concluded that the differences in image quality were so small that the authors were unable to recommend the use of 3.0‐T MRI units over 1.5‐T units or vice versa (Wardlaw et al., 2012). Therefore, it is unlikely that the differences between present findings and previous work are solely due to differences in magnetic field strength.

Finally, no information regarding illness subtype was collected, and eating disorder symptomatology and duration of illness were assessed through self‐report. Therefore, it is possible that a lack of certainty regarding age at illness onset or of insight into the illness may have affected the current findings. Future studies may benefit from including a clinician assessment of psychopathology or utilising a clinical interview to determine current symptomatology, illness subtype, and duration of illness.

## 6 CONCLUSIONS

The aim of the present study was to replicate and validate previous work examining alterations in cortical morphometry in people with AN. As hypothesised, we found the women with AN had significantly reduced global cortical volume, which replicated previous findings. Similarly, we found reduced local cortical gyrification in the women with AN indicating a reduction in the amount of cortical surface buried within the sulci in AN, which also replicated previous work. Contrary to our hypotheses, however, we were not able to replicate previous findings reporting widespread reduction in local cortical thickness and curvature. Instead, we found small clusters of reduced cortical thickness in the AN group, primarily located in the parietal and occipital cortices, and no significant group differences in local cortical curvature. We also found increased cortical thickness in the AN group in a small cluster in the ACC. Finally, we found group differences in local cortical surface area in small regional clusters in the frontal and temporal cortices, which is in direct contrast with previous finding of no significant group differences. The present findings validate previous work documenting alterations in global cortical volume and local gyrification in acute AN. Moreover, the present work highlights the need for further investigation of local cortical folding, thickness, and surface area in AN to gain further insight into the biological underpinnings of AN.

## CONFLICT OF INTEREST

None

## Supporting information

Table S1. Information about excluded participants
Table S2. Information regarding psychotropic medication
Table S3. Differences between the datasets in cortical volume
Table S4. Correlations between global cortical morphometry and eating disorder characteristics
Table S5. Impact of medication status on global cortical volume
Table S6. Relationship between global cortical volume and local cortical surface area
