# Supplementary material for: Cortical morphometry in anorexia nervosa: An out‐of‐sample replication study
Source: Eur Eat Disord Rev. 2019 Jun 6;27(5):507–20. doi: 10.1002/erv.2686 (PMC6698193; doi:10.1002/erv.2686)
Supplement: Supplementary file 1 — Table S1. Information about excluded participants Table S2. Information regarding psychotropic medication Table S3. Differences between the datasets in cortical volume Table S4. Correlations between global cortical morphometry and eating disorder characteristics Table S5. Impact of medication status on global cortical volume Table S6. Relationship between global cortical volume and local cortical surface area [file ERV-27-507-s001.docx]

Supplementary Table 1. Information about excluded participants

| ID | Group | Age (years) | BMI | EDEQ Total | Medication status | Duration of illness (years) | Handedness | Dataset | Reason for exclusion |
| --- | --- | --- | --- | --- | --- | --- | --- | --- | --- |
| 1 | HC | 25.0 | 23.8 | 1.1 | N/A | N/A | Left handed | 2 | Left handedness |
| 2 | HC | 29.0 | 21.0 | 0.3 | N/A | N/A | Left handed | 2 | Left handedness |
| 3 | AN | 30.0 | 15.9 | 3.0 | medicated | NR | Left handed | 2 | left handedness |
| 4 | HC | 23.0 | 19.5 | 0.2 | N/A | N/A | Left handed | 2 | left handedness |
| 5 | AN | 22.0 | 15.0 | 5.2 | medication-free | 5.0 | Left handed | 2 | left handedness |
| 6 | AN | 18.0 | 15.0 | 5.3 | medication-free | 4.0 | Left handed | 2 | left handedness |
| 7 | AN | 37.0 | 14.8 | 5.1 | medicated | 20.0 | Left handed | 2 | left handedness |
| 8 | AN | missing | missing | missing | missing | missing | missing | 1 | Substantial missing data |
| 9 | AN | missing | missing | missing | missing | missing | missing | 1 | Substantial missing data |
| 10 | AN | missing | missing | missing | missing | missing | missing | 1 | Substantial missing data |
| 11 | missing | missing | missing | missing | missing | missing | missing | 1 | Substantial missing data |
| 12 | missing | missing | missing | missing | missing | missing | missing | 1 | Substantial missing data |
| 13 | missing | missing | missing | missing | missing | missing | missing | 1 | Substantial missing data |
| 14 | missing | missing | missing | missing | missing | missing | missing | 1 | Substantial missing data |
| 15 | missing | missing | missing | missing | missing | missing | missing | 1 | Substantial missing data |
| 16 | AN | 22.0 | 19.1 | 1.5 | medicated | 6.0 | Right handed | 2 | Weight restored |
| 17 | AN | 19.0 | 19.5 | 5.0 | medicated | 5.0 | Right handed | 1 | Weight restored |
| 18 | AN | missing | 19.5 | missing | missing | missing | missing | 1 | Weight restored |

BMI= body mass index, EDEQ = Eating Disorders Examination Questionnaire, NR = not reported, AN = anorexia nervosa, HC = healthy comparison

Supplementary Table 2. Information regarding psychotropic medication

| Name of drug | N of AN participants taking the drug |
| --- | --- |
| Citalopram | 5 |
| Fluoxetine | 5 |
| Venlafaxine | 1 |
| Pregabalin | 1 |
| Quetiapine | 1 |
| Levothyroxine | 1 |
| Lamotrigine | 1 |

Information regarding the type of psychotropic medication was collected as part of Study 2 (Fonville et al., 2014) and was available for 13 AN participants.

Supplementary Table 3. Differences between the datasets in cortical volume

|  | Dataset 1 (N = 47)  Mean (SD)  Range  N | Dataset 2 (N = 53)  Mean (SD)  Range  N | t statistic, p-value |
| --- | --- | --- | --- |
| Age | 27.29 (7.78)  18.00 – 60.00  45 | 26.50 (6.26)  18.00 – 46.00  52 | t(95) = -0.55, p = 0.581 |
| BMI | 18.54 (3.22)  12.19 – 26.30  47 | 19.10 (3.50)  12.00 – 25.50  53 | t(98) = 0.82, p = 0.414 |
| EDEQ | 2.08 (1.97)  0.00 – 5.82  47 | 2.17 (1.86)  0.00 – 5.80  53 | t(96) = 0.22, p = 0.825 |
| Anxiety z-score | -0.05 (0.99)  -0.94 – 2.12  47 | -0.05 (0.99)  -1.45 – 1.74  53 | t(98) = 0.02, p = 0.984 |
| Depression z-score | -0.06 (0.98)  -0.93 – 2.21  47 | -0.04 (1.01)  -0.99 – 2.43  53 | t(98) = -0.10, p = 0.919 |
| Global cortical volume | 455304.32 (46630.96)  386641.8 – 582408.9  47 | 456416.24 (39268.11)  368184.0 – 573728.8  53 | t(98) = 0.13, p = 0.898 |

Dataset 1 = data from Study 1; Dataset 2 = data from Study 2; BMI = body mass index; EDEQ = Eating Disorders Examination Questionnaire; SD = standard deviation.

Supplementary Table 4. Correlations between global cortical morphometry and eating disorder characteristics

| Hemisphere | Measure | Cluster | EDEQ Total | BMI | Duration of illness (years) |
| --- | --- | --- | --- | --- | --- |
| Left & right | Cortical volume | N/A | r = 0.30, p = 0.046 | r = 0.11, p = 0.485 | r = -0.10, p = 0.521 |
| Left | Thickness | SPC | r = 0.37, p = 0.016 | r = 0.25, p = 0.109 | r = -0.22, p = 0.172 |
|  |  | Paracentral cortex | r = 0.30, p = 0.051 | r = 0.29, p = 0.060 | r = -0.14, p = 0.377 |
|  |  | ACC | r = -0.08, p = 0.603 | r = -0.06, p = 0.696 | r = -0.38, p = 0.016 |
| Right |  | SPC | r = 0.37, p = 0.013 | r = 0.32, p = 0.038 | r = -0.09, p = 0.590 |
|  |  | Precuneus | r = 0.23, p = 0.139 | r = 0.40, p = 0.008 | r = -0.05, p = 0.760 |
|  |  | LOC | r = 0.22, p = 0.154 | r = 0.19, p = 0.235 | r = 0.02, p = 0.882 |
| Left | LGI | LFC | r = 0.02, p = 0.899 | r = 0.15, p = 0.353 | r = -0.38, p = 0.017 |
|  |  | Postcentral cortex | r = -0.02, p = 0.889 | r = 0.25, p = 0.100 | r = -0.54, p = 0.0003 |
|  |  | STC | r = -0.01, p = 0.935 | r = 0.07, p = 0.670 | r = -0.32, p = 0.045 |
| Right |  | Insula | r = 0.02, p = 0.874 | r = 0.09, p = 0.567 | r = -0.23, p = 0.149 |
|  |  | LFC | r = 0.011, p = 0.492 | r = 0.18, p = 0.254 | r = -0.34, p = 0.034 |
|  |  | SMG | r = 0.16, p = 0.304 | r = 0.25, p = 0.110 | r = -0.43, p = 0.005 |
| Left | Surface area | SFC | r = 0.13, p = 0.393 | r = -0.07, p = 0.659 | r = -0.18, p = 0.256 |
|  |  | STC | r = 0.04, p = 0.777 | r = -0.14, p = 0.376 | r = 0.16, p = 0.336 |
|  |  | ITC | r = -0.12, p = 0.455 | r = -0.03, p = 0.834 | r = -0.21, p = 0.192 |

SPC = superior parietal cortex, ISFC = superior frontal cortex, LFC = lateral frontal cortex, SMG = supramarginal gyrus, LOC = lateral occipital cortex, ACC = anterior cingulate cortex, STC = superior temporal cortex, ITC = inferior temporal cortex, EDEQ = Eating Disorder Examination Questionnaire, BMI = body mass index, N/A = not applicable

Supplementary Table 5. Impact of medication status on global cortical volume

|  | Medicated AN (N = )  Mean (SD) | Non-medicated AN (N = )  Mean (SD) | t score, p-value |
| --- | --- | --- | --- |
| Cortical volume (mm^3^) | 447045.17 (32739.36) | 430152.51 (33746.60) | medication status: t(41) = 1.68, p = 0.100  dataset: t(41) = 1.06, p = 0.296 |

AN = anorexia nervosa, SD = standard deviation, mm^3^ = cubic millimetre

Supplementary Table 6. Relationship between global cortical volume and local cortical surface area

| Peak MNI coordinates | | | Cluster Z score | | Corrected CWP | Cluster size (mm^2^) | | Number of vertices | | Peak region | |
| --- | --- | --- | --- | --- | --- | --- | --- | --- | --- | --- | --- |
| X | Y | Z | |  |  |  | |  | |  |  |
| 9.1 | 12.7 | 63.9 | | 4.38 | 0.004 | | 182.97 | | 393 | | SFC |

SFC = superior frontal cortex, CWP = cluster-wise p-value, MNI = Montreal Neurological institute
